# Emergency department use by patients with end-stage renal disease in the United States

**DOI:** 10.1186/s12873-021-00420-8

**Published:** 2021-03-02

**Authors:** Ningyuan Wang, Jiao Pei, Hui Fan, Yaseen Ali, Anna Prushinskaya, Jian Zhao, Xingyu Zhang

**Affiliations:** 1 College of Literature, Science, and the Arts, University of Michigan, Ann Arbor, USA; 2 Sichuan Cancer Hospital & Institute, Sichuan Cancer Center, School of Medicine, University of Electronic Science and Technology of China, Chengdu, China; 3 Department of Epidemiology and Biostatistics, West China School of Public Health and West China Fourth Hospital, Sichuan University, Chengdu, China; 4 Department of Preventive Medicine, North Sichuan Medical College, Nanchong, China; 5 Department of Systems, Populations, and Leadership, University of Michigan School of Nursing, Ann Arbor, MI 48109 USA; 6 MRC Integrative Epidemiology Unit, University of Bristol, Bristol, UK; 7 NIHR Bristol Biomedical Research Centre, University of Bristol, Bristol, UK; 8 Population Health Sciences, Bristol Medical School, University of Bristol, Bristol, UK

**Keywords:** End-stage renal disease, Emergency medicine, National characteristics, Resource utilization

## Abstract

**Background:**

We sought to describe the national characteristics of ED visits by patients with end-stage renal disease (ESRD) in the United States in order to improve the emergency treatment and screening of ESRD patients.

**Methods:**

We analyzed data from 2014 to 2016 ED visits provided by the National Hospital Ambulatory Medical Care Survey. We sampled adult (age ≥ 18 years) ED patients with ESRD. By proportion or means of weighted sample variables, we quantified annual ED visits by patients with ESRD. We investigated demographics, ED resource utilization, clinical characteristics, and disposition of patients with ESRD and compared these to those of patients without ESRD. Logistic regression models were used to estimate the association between these characteristics and ESRD ED visits.

**Results:**

Approximately 722,692 (7.78%) out of 92,899,685 annual ED visits represented ESRD patients. Males were more likely to be ESRD patients than females (aOR: 1.34; 95% CI: 1.09–1.66). Compare to whites, non-Hispanic Blacks were 2.55 times more likely to have ESRD (aOR: 2.55; 95% CI: 1.97–3.30), and Hispanics were 2.68 times more likely to have ESRD (95% CI: 1.95–3.69). ED patients with ESRD were more likely to be admitted to the hospital (aOR: 2.70; 95% CI: 2.13–3.41) and intensive care unit (ICU) (aOR: 2.21; 95% CI: 1.45–3.38) than patients without ESRD. ED patients with ESRD were more likely to receive blood tests and get radiology tests.

**Conclusion:**

We described the unique demographic, socioeconomic, and clinical characteristics of ED patients with ESRD, using the most comprehensive, nationally representative study to date. These patients’ higher hospital and ICU admission rates indicate that patients with ESRD require a higher level of emergency care.

**Supplementary Information:**

The online version contains supplementary material available at 10.1186/s12873-021-00420-8.

## Background

Kidney diseases are the ninth leading cause of death in the United States. Fifteen percent of U.S. adults, about 37 million people, were estimated to have chronic kidney disease (CKD) in 2019 [1]. End-stage renal disease (ESRD), the final stage of CKD, has emerged as one of the most important public health concerns in the United States. In 2016, approximately 125,000 people in the U.S. started treatment for end-stage kidney disease, and over 726,000 were on dialysis or living with a kidney transplant [1]. The total Medicare expenditure (excluding prescription drugs) for patients with ESRD or kidney failure reached $35 billion, accounting for about 7% of the Medicare paid claims costs [2]. At the end of 2017, 746,557 ESRD cases were reported in the U.S., which represents an increase of 2.6% from 2016 and an increase of 91.1% from 2000 [3]. The prevalence of ESRD in the U.S. is expected to continue to increase through the year 2030 [4].

Patients with ESRD are often frail and comorbid which puts them at high risk for emergency department (ED) visits and hospitalization. Nearly two-thirds of patients with ESRD are admitted to the hospital in the year prior to initiating renal replacement therapy [5], and the rehospitalization rate for patients with ESRD is more than 30% higher than the rate of rehospitalization for other patients [6, 7]. The ED plays an important role for ESRD patients that seek urgent care. Therefore, identifying the characteristics of ESRD patients who visit the ED would be beneficial and can help to optimize ED resource utilization and perhaps alleviate the burden currently placed on the ED by these patients. Previous studies have documented high hospital resource use among patients with ESRD [8, 9]; however, literature focused on ED utilization amongst this population is sparse, and specifically literature that relates ESRD visits proportionally to other ED visits is limited [10]. As such, in the present study, we aim to examine the national characteristics of ED utilization among patients with ESRD and their corresponding usage proportion in the U.S. from 2014 to 2016.

## Methods

### Study population

The study population consists of all adult patients (age ≥ 18 years) (*N* = 42,832; Weighted *N* = 278,699,057) in the National Hospital Ambulatory Medical Care Survey Emergency Department Subfile (NHAMCS-ED) from 2014 to 2016 [11]. NHAMCS-ED is a nationally representative, multistage, stratified probability sample of ED visits in the United States administered by the National Center for Health Statistics, a branch of the Centers for Disease Control and Prevention. The NHAMCS-ED sample is collected from approximately 300 hospital-based EDs per year, which are randomly selected from approximately 1900 geographic areas in all 50 states. The survey uses a standardized data collection form to gather detailed information from approximately 100 patients per hospital-based ED.

### Study variables

The primary outcome for the study is the patient ESRD status noted as “ESRD status.” In NHAMCS, ESRD status “includes all types of end-stage renal disease and chronic kidney failure due to diabetes or hypertension” [12].

The secondary outcomes include the emergency severity index (ESI) score (a five-level ED triage algorithm providing clinically relevant stratification of patients into five groups from 1 (most urgent) to 5 (least urgent) on the basis of acuity and resource needs); hospital admission; intensive care unit (ICU) admission; blood tests; imaging (including X-ray, CT, ultrasound, MRI); procedures (BiPAP/CPAP; bladder catheter; cast, splint, wrap; central line; IV fluids; CPR; endotracheal intubation; incision & drainage (I&D); IV fluids; lumbar puncture (LP); nebulizer therapy; pelvic exam; skin adhesives; suturing/staples; Other); whether the patient left before triage/treatment; length of stay; and whether the patient died in the ED/hospital.

The covariates examined include demographic characteristics (e.g., patient age, sex, race/ethnicity, region, residence type); time, date, and mode of arrival; insurance status; triage vital signs (including temperature, pain scale, blood pressure, etc.), and reasons for ED visit.

### Statistical analysis

Population characteristics between ESRD and non-ESRD groups were described and compared using chi-square or *t* test. We used logistic regression to examine the association between the primary outcome (ED patients with ESRD versus ED patients without ESRD) and the covariates. We also used logistic regression to test the association between ESRD status and secondary outcomes by adjusting for other covariates. Missing values were imputed with the median of each covariate when establishing the multivariable logistic regression. SAS (version 9.4) was used for analyses, with *α* = 0.05 set as the statistical significance threshold. This study was determined to be exempt by the institutional review board.

## Results

Between 2014 and 2016, there were 278,699,057 total adult ED visits in the United States. Patients with ESRD made up approximately 2,168,075 (7.78% or 722,692 annually) of these visits. In addition, the proportion of ED visits by patients with ESRD increased between 2014 to 2016. Basic characteristics are described in Table 1. The proportion of ED visits by patients with ESRD varied by US census region: Northwest, 13.4%; Midwest, 19.8%; South, 45.6%; and West, 21.2% (*p* < 0.01). ESRD patients and non-ESRD patients differed significantly in age and race (*p* < 0.001).

**Table 1:** Baseline characteristics of patients presenting to the ED, stratified by ESRD, NHAMCS 2014–2016

	Unweighted sample			Weighted sample			*p* value
	All	No ESRD	ESRD	All	No ESRD	ESRD	
	42,832	42,465	367	278,699,057	276,530,981	2,168,075	
Male	18,469 (43.1)	18,283 (43.1)	186 (50.7)	119,751,766 (43.0)	118,611,308 (42.9)	1,140,459 (52.6)	0.0033
Age							
18–39	17,912 (41.8)	17,862 (42.1)	50 (13.6)	118,068,691 (42.4)	117,768,064 (42.6)	300,627 (13.9)	< 0.001
40–49	6662 (15.6)	6629 (15.6)	33 (9.0)	43,185,040 (15.5)	43,021,286 (15.6)	163,755 (7.6)	
50–59	6707 (15.7)	6638 (15.6)	69 (18.8)	42,679,091 (15.3)	42,215,775 (15.3)	463,316 (21.4)	
60–74	6678 (15.6)	6542 (15.4)	136 (37.1)	43,420,164 (15.6)	42,634,581 (15.4)	785,583 (36.2)	
> =75	4873 (11.4)	4794 (11.3)	79 (21.5)	31,346,071 (11.2)	30,891,277 (11.2)	454,793 (21.0)	
Race/ethnicity							
White	27,251 (63.6)	27,079 (63.8)	172 (46.9)	175,775,546 (63.1)	174,659,617 (63.2)	1,115,929 (51.5)	< 0.001
Black	9207 (21.5)	9092 (21.4)	115 (31.3)	62,663,628 (22.5)	62,051,038 (22.4)	612,590 (28.3)	
Hispanic	5152 (12.0)	5094 (12.0)	58 (15.8)	33,391,671 (12.0)	33,055,349 (12.0)	336,322 (15.5)	
Asian	804 (1.9)	793 (1.9)	11 (3.0)	4,392,213 (1.6)	4,349,798 (1.6)	42,415 (2.0)	
Other	418 (1.0)	407 (1.0)	11 (3.0)	2,475,999 (0.9)	2,415,180 (0.9)	60,819 (2.8)	
Residence type							
Private residence	39,819 (95.1)	39,498 (95.1)	321 (89.7)	258,354,513 (95.3)	256,528,244 (95.3)	1,826,269 (85.6)	< 0.001
Nursing home	885 (2.1)	856 (2.1)	29 (8.1)	5,875,161 (2.2)	5,632,038 (2.1)	243,123 (11.4)	
Homeless	534 (1.3)	534 (1.3)	0 (0.0)	2,480,109 (0.9)	2,480,109 (0.9)	0 (0.0)	
Other	651 (1.6)	643 (1.5)	8 (2.2)	4,501,686 (1.7)	4,437,115 (1.6)	64,571 (3.0)	
Insurance type							
Private insurance	12,446 (30.8)	12,411 (31.0)	35 (9.8)	79,443,111 (30.5)	79,249,592 (30.7)	193,519 (9.1)	< 0.001
Medicare	10,517 (26.0)	10,278 (25.7)	239 (66.8)	66,956,323 (25.7)	65,443,229 (25.3)	1,513,093 (71.2)	
Medicaid or CHIP	11,148 (27.6)	11,080 (27.7)	68 (19.0)	71,529,605 (27.5)	71,197,275 (27.6)	332,331 (15.6)	
Uninsured	4886 (12.1)	4876 (12.2)	10 (2.8)	33,248,283 (12.8)	33,203,302 (12.8)	44,981 (2.1)	
Other	1406 (3.5)	1400 (3.5)	6 (1.7)	9,371,908 (3.6)	9,329,217 (3.6)	42,691 (2.0)	
Arrive by ambulance	7729 (18.5)	7600 (18.4)	129 (35.8)	49,769,047 (18.3)	48,948,071 (18.2)	820,977 (38.3)	< 0.001
Seen within last 72 h	1914 (4.9)	1898 (4.9)	16 (4.8)	11,953,039 (4.8)	11,874,648 (4.8)	78,391 (4.1)	0.8948
Pain level							
No pain	7711 (24.4)	7610 (24.2)	101 (39.8)	46,478,004 (23.1)	45,940,926 (23.0)	537,078 (37.6)	< 0.001
Mild	2916 (9.2)	2903 (9.2)	13 (5.1)	18,235,636 (9.1)	18,178,674 (9.1)	56,962 (4.0)	
Moderate	9430 (29.8)	9363 (29.8)	67 (26.4)	60,509,861 (30.1)	60,090,165 (30.1)	419,696 (29.4)	
Severe	11,602 (36.6)	11,529 (36.7)	73 (28.7)	75,762,102 (37.7)	75,347,113 (37.8)	414,989 (29.0)	
Temperature							
36 °C–38 °C	38,083 (94.6)	37,784 (94.7)	299 (90.6)	249,171,894 (95.1)	247,406,971 (95.1)	1,764,922 (92.5)	< 0.001
< =36 °C	1522 (3.8)	1504 (3.8)	18 (5.5)	9,089,224 (3.5)	9,001,036 (3.5)	88,187 (4.6)	
> 38 °C	635 (1.6)	622 (1.6)	13 (3.9)	3,863,922 (1.5)	3,808,689 (1.5)	55,233 (2.9)	
Heart Rate							
< =90	28,489 (66.5)	28,242 (66.5)	247 (67.3)	184,822,552 (66.3)	183,317,566 (66.3)	1504,986 (69.4)	0.5082
90–100	7169 (16.7)	7109 (16.7)	60 (16.3)	46,314,663 (16.6)	45,999,951 (16.6)	314,712 (14.5)	
100–110	3906 (9.1)	3876 (9.1)	30 (8.2)	25,427,295 (9.1)	25,268,229 (9.1)	159,066 (7.3)	
110–120	1988 (4.6)	1974 (4.6)	14 (3.8)	13,118,183 (4.7)	13,062,583 (4.7)	55,600 (2.6)	
> 120	1280 (3.0)	1264 (3.0)	16 (4.4)	9,016,363 (3.2)	8,882,652 (3.2)	133,711 (6.2)	
DBP							
60–80	19,358 (45.2)	19,213 (45.2)	145 (39.5)	125,677,278 (45.1)	124,830,342 (45.1)	846,937 (39.1)	< 0.001
< 60	4312 (10.1)	4233 (10.0)	79 (21.5)	26,198,088 (9.4)	25,714,760 (9.3)	483,328 (22.3)	
> 80	19,162 (44.7)	19,019 (44.8)	143 (39.0)	126,823,690 (45.5)	125,985,881 (45.6)	837,810 (38.6)	
SBP							
80–120	9773 (22.8)	9687 (22.8)	86 (23.4)	61,351,488 (22.0)	60,857,637 (22.0)	493,851 (22.8)	0.4365
< 80	1588 (3.7)	1570 (3.7)	18 (4.9)	9,419,022 (3.4)	9,310,953 (3.4)	108,068 (5.0)	
> 120	31,471 (73.5)	31,208 (73.5)	263 (71.7)	207,928,547 (74.6)	206,362,392 (74.6)	1,566,155 (72.2)	
Census Region							
Northeast	7176 (16.8)	7140 (16.8)	36 (9.8)	43,967,048 (15.8)	43,675,459 (15.8)	291,588 (13.4)	0.0004
Midwest	10,893 (25.4)	10,807 (25.4)	86 (23.4)	74,304,118 (26.7)	73,875,207 (26.7)	428,911 (19.8)	
South	15,430 (36.0)	15,268 (36.0)	162 (44.1)	105,760,507 (37.9)	104,771,742 (37.9)	988,765 (45.6)	
West	9333 (21.8)	9250 (21.8)	83 (22.6)	54,667,385 (19.6)	54,208,574 (19.6)	458,811 (21.2)	
This visit is related to							
Injury/trauma	12,286 (30.1)	12,248 (30.3)	38 (11.0)	78,178,483 (29.5)	77,992,283 (29.6)	186,200 (9.1)	< 0.001
Overdose/poisoning	499 (1.2)	498 (1.2)	1 (0.3)	3,358,380 (1.3)	3,349,593 (1.3)	8787 (0.4)	
Adverse effect of medical/surgical treatment	1099 (2.7)	1063 (2.6)	36 (10.4)	7,170,683 (2.7)	6,961,906 (2.6)	208,777 (10.2)	
Visit not related to any above	26,692 (65.4)	26,424 (65.3)	268 (77.7)	174,903,611 (66.0)	173,277,339 (65.9)	1,626,272 (79.4)	
Questionable injury status	214 (0.5)	212 (0.5)	2 (0.6)	1,546,669 (0.6)	1,528,096 (0.6)	18,573 (0.9)	

Tables 2, 3, and 4 describe the proportions and associations of ESI, hospital admission, ICU admission, and medical resource utilization for ESRD and non-ESRD patients. The hospital admission rate among ED patients was 2.70 times higher for patients with ESRD (95% CI: 2.13–3.41); ESRD patients were also 4.72 times more likely to receive immediate or emergent vs. semi- or non-urgent ESI scores compared to patients without ESRD (95% CI: 3.00–7.41). The ICU admission rate was 2.21 times higher for patients with ESRD (95% CI: 1.45–3.38). ED patients with ESRD were 2.54 times more likely to receive blood tests (95% CI: 1.89–3.40) as well as more likely to utilize X-rays (95% CI: 1.43–2.24).

**Table 2:** Selected reason for visit and emergency department diagnosis among ED patients with ESRD, NHAMCS 2014–2016

	Unweighted sample			Weighted sample		
	All	No ESRD	ESRD	All	No ESRD	ESRD
Reason for visit						
General Symptoms	8187 (19.1)	8069 (19.0)	118 (32.2)	53,664,580 (19.3)	52,934,900 (19.2)	729,680 (33.7)
Symptoms Referable to Psychological and Mental Disorders	1700 (4.0)	1687 (4.0)	13 (3.5)	9,426,523 (3.4)	9,331,220 (3.4)	95,303 (4.4)
Symptoms Referable to the Nervous System	3304 (7.7)	3279 (7.7)	25 (6.8)	20,833,741 (7.5)	20,708,936 (7.5)	124,805 (5.8)
Symptoms Referable to the Cardiovascular and Lymphatic Systems	889 (2.1)	877 (2.1)	12 (3.3)	5,993,917 (2.2)	5,914,527 (2.1)	79,390 (3.7)
Symptoms Referable to the Eyes and Ears	848 (2.0)	847 (2.0)	1 (0.3)	5,778,778 (2.1)	5,772,695 (2.1)	6083 (0.3)
Symptoms Referable to the Respiratory System	4198 (9.8)	4135 (9.8)	63 (17.2)	27,856,021 (10.0)	27,508,840 (10.0)	347,181 (16.0)
Symptoms Referable to the Digestive System	6807 (15.9)	6758 (15.9)	49 (13.4)	46,038,272 (16.5)	45,725,454 (16.6)	312,819 (14.4)
Symptoms Referable to the Genitourinary System	2477 (5.8)	2462 (5.8)	15 (4.1)	14,984,361 (5.4)	14,913,890 (5.4)	70,470 (3.3)
Symptoms Referable to the Skin, Nails, and Hair	1333 (3.1)	1328 (3.1)	5 (1.4)	8,716,118 (3.1)	8,690,203 (3.1)	25,915 (1.2)
Symptoms Referable to the Musculoskeletal System	6519 (15.2)	6493 (15.3)	26 (7.1)	42,820,579 (15.4)	42,682,302 (15.5)	138,277 (6.4)
Other	6501 (15.2)	6461 (15.2)	40 (10.9)	42,147,135 (15.1)	41,908,983 (15.2)	238,152 (11.0)

**Table 3:** Proportion of emergency severity index, hospital admission, ICU admission, medical resources utilization, stratified by ESRD, NHAMCS 2014–2016

	Unweighted sample			Weighted sample			
	All	No ESRD	ESRD	All	No ESRD	ESRD	*p* value
ESI score							
Immediate	239 (0.8)	235 (0.8)	4 (1.5)	1,496,327 (0.8)	1,471,879 (0.7)	24,448 (1.7)	< 0.001
Emergent	3615 (11.6)	3529 (11.5)	86 (32.8)	23,433,327 (11.8)	22,976,847 (11.7)	456,480 (31.6)	
Urgent	15,392 (49.5)	15,248 (49.5)	144 (55.0)	97,000,149 (49.0)	96,286,096 (49.0)	714,053 (49.4)	
Semi-urgent	10,051 (32.3)	10,034 (32.6)	17 (6.5)	65,085,335 (32.9)	64,950,854 (33.0)	134,480 (9.3)	
Non-urgent	1784 (5.7)	1773 (5.8)	11 (4.2)	11,046,598 (5.6)	10,931,909 (5.6)	114,689 (7.9)	
Hospital Admission	5852 (13.7)	5695 (13.4)	157 (42.8)	36,388,538 (13.1)	35,517,254 (12.8)	871,284 (40.2)	< 0.001
ICU	698 (1.6)	669 (1.6)	29 (7.9)	4,647,353 (1.7)	4,506,861 (1.6)	140,492 (6.5)	< 0.001
In hospital death	201 (0.5)	192 (0.5)	9 (2.5)	1,342,510 (0.5)	1,298,220 (0.5)	44,290 (2.0)	< 0.001
Left before/after triage	1085 (2.5)	1076 (2.5)	9 (2.5)	6,792,175 (2.4)	6,722,799 (2.4)	69,376 (3.2)	0.9212
Blood test	21,958 (51.3)	21,654 (51.0)	304 (82.8)	142,656,097 (51.2)	140,833,111 (50.9)	1,822,986 (84.1)	< 0.001
Any image	21,950 (51.2)	21,709 (51.1)	241 (65.7)	144,824,612 (52.0)	143,375,099 (51.8)	1,449,513 (66.9)	< 0.001
X-ray	15,099 (35.3)	14,894 (35.1)	205 (55.9)	99,429,274 (35.7)	98,179,495 (35.5)	1,249,778 (57.6)	< 0.001
CT	8414 (19.6)	8338 (19.6)	76 (20.7)	54,986,804 (19.7)	54,559,942 (19.7)	426,863 (19.7)	0.6063
Ultrasound	2218 (5.2)	2205 (5.2)	13 (3.5)	14,936,538 (5.4)	14,833,060 (5.4)	103,478 (4.8)	0.1554
MRI	446 (1.0)	438 (1.0)	8 (2.2)	2,831,626 (1.0)	2,791,440 (1.0)	40,186 (1.9)	0.0309
Other Imaging	604 (1.4)	595 (1.4)	9 (2.5)	4,297,097 (1.5)	4,239,282 (1.5)	57,815 (2.7)	0.0890
Procedure	21,021 (49.1)	20,807 (49.0)	214 (58.3)	133,801,012 (48.0)	132,620,938 (48.0)	1,180,074 (54.4)	0.0011
Waiting time (minutes, MEANS (95% CI))	41.1 (40.3–41.8)	41.0 (40.3–41.8)	45.2 (37.7–52.7)	39.9 (39.2–40.6)	39.9 (39.1–40.6)	46.5 (38.8–54.1)	0.7500
Length of visit (minutes, MEANS (95% CI))	245.6 (241.6–249.6)	244.1 (240.1–248.1)	422.7 (339.2–506.3)	230.2 (226.7–233.8)	228.7 (225.2–232.2)	450.3 (358.0–542.5)	<.0001

Notes: Waiting time: time from arrival to seeing the physician. Length of visit: time from arrival to discharge

**Table 4:** Odds ratio of emergency severity index, hospital admission, ICU admission, medical resources utilization for ESRD vs. non-ESRD patients, NHAMCS 2014–2016

	Crude odds ratio	Adjusted for		
		Demographics	+ Social economic	+ Visiting & Clinical
ESI Score: Immediate or Emergent vs. Semi- or Non-Urgent	10.07 (6.58–15.41)	6.98 (4.53–10.74)	6.74 (4.37–10.38)	4.72 (3.00–7.41)
ESI Score: Urgent vs. Semi- or Non-Urgent	3.98 (2.65–5.96)	3.33 (2.22–5.01)	3.24 (2.15–4.87)	2.46 (1.62–3.74)
Hospital Admission	4.83 (3.92–5.95)	3.32 (2.67–4.13)	3.30 (2.65–4.11)	2.70 (2.13–3.41)
ICU	5.36 (3.64–7.90)	3.25 (2.20–4.82)	3.07 (2.06–4.58)	2.21 (1.45–3.38)
Death	5.54 (2.81–10.89)	3.03 (1.53–6.01)	2.65 (1.33–5.30)	1.64 (0.76–3.55)
Left	0.97 (0.50–1.88)	1.23 (0.63–2.40)	1.08 (0.55–2.11)	0.93 (0.47–1.82)
Blood test	4.64 (3.53–6.09)	3.60 (2.73–4.74)	3.41 (2.59–4.49)	2.54 (1.89–3.40)
Any imaging	1.83 (1.47–2.27)	1.36 (1.09–1.70)	1.36 (1.09–1.70)	1.37 (1.09–1.72)
X-ray	2.34 (1.90–2.88)	1.73 (1.40–2.14)	1.69 (1.36–2.09)	1.79 (1.43–2.24)
CT	1.07 (0.83–1.38)	0.80 (0.62–1.03)	0.81 (0.63–1.05)	0.80 (0.61–1.05)
Ultrasound	0.67 (0.39–1.17)	0.86 (0.49–1.51)	0.88 (0.50–1.55)	0.84 (0.47–1.48)
MRI	2.14 (1.06–4.34)	1.58 (0.78–3.22)	1.66 (0.81–3.39)	1.92 (0.93–4.00)
Procedure	1.33 (1.08–1.63)	1.24 (1.01–1.52)	1.24 (1.01–1.52)	1.20 (0.97–1.47)

Note: ^*^ + Demographics include: gender, age group, race/ethnicity; +Social economic: residence type, insurance type, census region; + Visiting & Clinical: year, week of day, arrive by ambulance, seen within last 72 h, pain level, temperature, heart rate, dialytic blood pressure, injury status, reason for visit

The associations between ED patients’ demographic, socioeconomic, and clinical characteristics and their ESRD status are outlined in Supplement Table 1. Male ED patients were 34% more likely to have ESRD than were female patients (aOR: 1.34; 95% CI: 1.09–1.66). Among ED patients, non-Hispanic Blacks were 2.55 times more likely than whites to have ESRD (aOR: 2.55; 95% CI: 1.97–3.30); Hispanics were 2.68 times more likely than whites to have ESRD (95% CI: 1.95–3.69); and Asians were 2.90 times more likely than whites to have ESRD (95% CI: 1.53–5.50).

Compared to ED patients inhabiting a private residence, those who were living in nursing homes were 1.53 times more likely to be ESRD patients (95% CI: 1.00–2.34). Compared to ED patients with private insurance, those with Medicare and Medicaid or CHIP were 4.23 and 2.05 times more likely to have ESRD (95% CI: 2.89–6.19, CI: 1.35–3.12, respectively).

Regarding vital signs, compared to patients with a DBP of 60–80, ED patients with DBP lower than 60 were 1.92 times to be ESRD patients (95% CI: 1.44–2.56). Compared to patients who arrived at the ED by other means, patients who arrived by ambulance were 1.58 times more likely to have ESRD (95% CI: 1.24–2.01). Meanwhile, ED patients who presented with an adverse effect of medical/surgical treatment were 6.58 times more likely to have ESRD than those presenting with injury or trauma (95% CI: 4.07–10.64).

## Discussion

ESRD is a complex clinical condition caused by chronic kidney disease, high blood pressure, and others, and the incidence of ESRD increases sharply with age in both sexes [13]. ESRD patients need special and professional health care in both emergency and non-emergency cases. Additionally, diabetes and hypertension account for more than 50% of cases of ESRD, and care of these patients increasingly depends on primary care physicians [14]. To our knowledge, this study is a representative large–scale study describing national characteristics of ED visits by ESRD patients. A thoughtful study by Lovasik et al. [15] examined the use of the ED among ESRD patients with Medicare. However, the population of their study was limited to ED patients with Medicare only, and the analysis of the study was around the characteristics of hospitalization. Our study focuses on all ED adult patient visits between 2014 and 2016 in the United States, and the study conclusions were drawn from a comparison of ED visits by patients with ESRD and non-ESRD status. In addition, our study also provides medical resource utilization information related to ED visits by ESRD patients, such as use the of blood tests and medical imaging in this population. This more extensive characterization helps generate nationally-representative results about ED visits by ESRD patients. Another ED utilization analysis by Ronksley et al. [16] was national in scope but explored ED use among patients with CKD rather than ESRD, whereas the focus of the present study is ESRD.

From 2014 to 2016, 2,168,075 ESRD patients visited the ED in total, and the number of annual visits by those patients has increased stably. Demographic factors were associated with the prevalence of ESRD in ED patients. One important demographic factor is age. Our study analysis suggests that compared to patients who visit the ED and do not have ESRD, ESRD patients who visit the ED are more likely to be senior patients. This increased likelihood of older age makes sense within the context of other trends. For example, nearly half of incident dialysis patients in the United States annually are senior citizens [17]. Age alone increases the risk of mortality in ESRD patients [18]. And, in addition to being an independent risk factor for increased mortality in patients with ESRD, increased age carries further risk because aging is also associated with cardiovascular disease. The cardiovascular mortality rate in ESRD patients is 10 to 20 times higher than that rate in the general population [19]. Therefore, clinical care of cardiovascular disease among these older ED patients with ESRD is necessary. As a result of these increased risks associated with age, we can expect that these older ESRD patients may require more extensive use of ED resources.

Another important demographic factor is gender. Our study suggests that compared to patients who visit the ED and do not have ESRD, ESRD patients who visit the ED are more likely to be male. This same gender difference in ESRD patients has been documented in the field of nephrology. For example, a nationwide survey of ESRD by the Japanese Society for Dialysis Therapy revealed a higher incidence and prevalence of ESRD in men, according to their research on gender differences in chronic kidney disease [20]. Some studies have found that women with ESRD have a reduced mortality risk [21], while others have found that this mortality risk advantage is diminished when assessing the risk of mortality in men and women who are on hemodialysis [22]. Further research into the complex interactions between gender and ESRD status is needed in order to understand how the increased proportion of male ESRD patients in the ED can translate into adjustments to clinical decision making in the ED.

Our study suggests that the ED visits prevalence among ESRD patients is significant higher in the South. Previous studies have assessed for geographic differences in ESRD incidence in the U.S. Rosansky et al. (1990) [23] found that ESRD treatment rates varied regionally across the U.S. after adjusting for race, sex, and age differences with very high rates in the southwestern states. Similarly, Foxman et al. (1991) [24], found regional variation across U.S. states with the highest ESRD incidence in the Southwest as well as the Southeast. Tanner et al. (2013) [25] focused on geographic variation in the prevalence of CKD and found that differences in CKD prevalence did not explain geographic variation in ESRD prevalence.

Another important difference revealed in the data is the differences in presenting vital signs between ESRD and non-ESRD patients. Patients with ESRD were more likely to have reduced blood pressure than were patients without ESRD. These alterations of vital signs were related to the adverse effect of medical/surgical treatment, which was the most likely reason for ED visits by ESRD patients. Presentation with alterations in vital signs may be related to outcomes such as increased return visits to the ED, increased rates of readmission, and increased need for higher level of care [26].

Finally, our analysis reveals several other indicators that patients of ESRD may be more complex and higher acuity than other patients. For example, in this study, we found that ED visits by ESRD patients were 1.5 times more likely to be from nursing homes than from a private residence, and that these patients are also more likely to be delivered by ambulance rather than by other means. This is consistent with previous findings that show that receiving hemodialysis in the post-dialysis initiation period was a high-risk time for falls among older adults [27].

Additionally, compared to non-ESRD patients, those with ESRD had higher rates of hospital and ICU admission. The higher rate of revisiting the ED as well as the higher rate of hospital admission in ESRD patients can be associated with higher severity of the condition, poor outcomes of previous treatments, and high costs. Many previous studies have similar findings. For example, the U.S. Renal Data System reported that an overall rehospitalization rate for patients with ESRD was 34% within 30 days of discharge [28].

Understanding the above characteristics of ED visits by ESRD patients may help the clinicians understand these patients who are at high risk for ED visit, hospital admission, and other health outcomes, as well as the need for increased medical resource use. As a result, clinicians can aim to improve the efficiency of clinical care and reduce the high rates of hospital admission, which in turn would not only benefit ESRD patients but also benefit hospitals in terms of better resource allocation and better financial allocation.

### Limitations

In the patient histories documented in the NHAMCS-ED data, patients are coded as either having or not having ESRD status, but information such as duration and treatment history were not tracked in the dataset. This information would help to better predict ESRD status among ED patients. As Iseki noted, ESRD is not a specific disease entity, but rather provides a framework for the consideration of treatment options [13]. Understanding the relationship between ESRD and other chronic diseases would help to determine risk factors for utilizing ED resources for ESRD patients. Another limitation is that the dataset did not provide information about patients’ other health conditions.

## Conclusions

This study enhanced the understanding of clinical characteristics of ED utilization in patients with ESRD. The study describes the characteristics of ESRD patients who visit the ED on a national scale. We found that there are gender, age, and racial/ethnic differences between ED patients with and without ESRD. ESRD patients are also more likely to present with alterations in vitals signs. Also, patients with ESRD are more likely to return to the ED, more likely to visit the ED due to complications of therapy, more likely to reside in a nursing home, and more likely to arrive by ambulance compared to non-ESRD patients. The above findings suggest that patients with ESRD have a higher demands for utilizing ED care and resources.

## Supplementary Information

**Additional file 1: Supplement Table 1.** Association between ED visiting with ESRD and patient visiting characteristics, NHAMCS 2014–2016. Note: the adjusted OR was from a logistic regression including all variables in the table.

## Data Availability

The NHAMCS-ED dataset can be accessed through the website of the US Centers for Disease Control and Prevention (CDC) (https://www.cdc.gov/nchs/ahcd/index.htm). The detailed explanation of the survey data for each year and the code book can be found here: https://ftp.cdc.gov/pub/Health_Statistics/NCHS/dataset_documentation/nhamcs/

## Abbreviations

aOR: Adjusted odds ratio
CI: Confidential interval
ED: Emergency department
NHAMCS-ED: National Hospital Ambulatory Medical Care Survey ED Subfile
ESI: Emergency severity index
ICU: intensive care unit
CT: Computed tomography
MRI: Magnetic resonance imaging
ESRD: End-stage renal disease
