# Supplementary material for: Emergency department use by patients with end-stage renal disease in the United States
Source: BMC Emerg Med. 2021 Mar 2;21:25. doi: 10.1186/s12873-021-00420-8 (PMC7927369; doi:10.1186/s12873-021-00420-8)
Supplement: Supplementary file 1 — Additional file 1: Supplement Table 1. Association between ED visiting with ESRD and patient visiting characteristics, NHAMCS 2014–2016. Note: the adjusted OR was from a logistic regression including all variables in the table. [file 12873_2021_420_MOESM1_ESM.docx]

Supplement Table 1. Association between ED visiting with ESRD and patient visiting characteristics, NHAMCS 2014–2016

| Effect | Crude OR (95% CI) | Adjusted OR (95% CI) |
| --- | --- | --- |
| Age |  |  |
| 18–39 | Reference [1] | Reference [1] |
| 40–49 | 1.78(1.15-2.76) | 1.62(1.04-2.54) |
| 50–59 | 3.71(2.58-5.35) | 2.93(2.00-4.28) |
| 60–74 | 7.43(5.36-10.28) | 3.57(2.46-5.17) |
| >=75 | 5.89(4.13-8.40) | 2.18(1.42-3.33) |
| Male vs Female | 1.36(1.11-1.67) | 1.34(1.09-1.66) |
| Race/ethnicity |  |  |
| White | Reference [1] | Reference [1] |
| Black | 1.99(1.57-2.53) | 2.55(1.97-3.30) |
| Hispanic | 1.79(1.33-2.42) | 2.68(1.95-3.69) |
| Asian | 2.18(1.18-4.03) | 2.90(1.53-5.50) |
| Other | 4.26(2.30-7.89) | 5.60(2.92-10.74) |
| Residence type |  |  |
| Private residence | Reference [1] | Reference [1] |
| Nursing home | 4.15(2.82-6.10) | 1.53(1.00-2.34) |
| Homeless | - | - |
| Other | 1.52(0.75-3.09) | 1.08(0.52-2.23) |
| Insurance type |  |  |
| Private insurance | Reference [1] | Reference [1] |
| Medicare | 6.93(4.86-9.88) | 4.23(2.89-6.19) |
| Medicaid or CHIP | 2.18(1.45-3.27) | 2.05(1.35-3.12) |
| Uninsured | 0.73(0.36-1.47) | 0.80(0.39-1.63) |
| Other | 1.52(0.64-3.62) | 1.39(0.58-3.38) |
| Temperature |  |  |
| 36 °C–38 °C | Reference [1] | Reference [1] |
| <=36 °C | 1.44(0.89-2.32) | 1.32(0.81-2.15) |
| >38 °C | 2.51(1.43-4.39) | 1.61(0.90-2.91) |
| Heart Rate |  |  |
| <=90 | Reference [1] | Reference [1] |
| 90–100 | 0.97(0.73-1.28) | 1.15(0.86-1.54) |
| 100–110 | 0.89(0.61-1.30) | 1.01(0.69-1.50) |
| 110–120 | 0.81(0.47-1.39) | 0.97(0.55-1.69) |
| >120 | 1.45(0.87-2.41) | 1.29(0.76-2.20) |
| DBP |  |  |
| 60–80 | Reference [1] | Reference [1] |
| <60 | 2.47(1.88-3.26) | 1.92(1.44-2.56) |
| >80 | 1.00(0.79-1.26) | 1.07(0.84-1.36) |
| Pain level |  |  |
| No pain | Reference [1] | Reference [1] |
| Mild | 0.34(0.19-0.60) | 0.57(0.31-1.02) |
| Moderate | 0.66(0.52-0.85) | 0.92(0.72-1.19) |
| Severe | 0.48(0.35-0.65) | 0.82(0.59-1.14) |
| 72 hours revisit vs not | 1.03(0.62-1.70) | 1.13(0.67-1.89) |
| Ambulance arrival vs not | 2.49(2.00-3.09) | 1.58(1.24-2.01) |
| Census Region |  |  |
| Northeast | Reference [1] | Reference [1] |
| Midwest | 1.58(1.07-2.33) | 1.60(1.08-2.39) |
| South | 2.10(1.47-3.02) | 2.02(1.39-2.93) |
| West | 1.78(1.20-2.64) | 1.77(1.18-2.65) |
| Reason for visit |  |  |
| General Symptoms | Reference [1] | Reference [1] |
| Symptoms Referable to Psychological and Mental Disorders | 0.53(0.30-0.94) | 0.56(0.31-1.00) |
| Symptoms Referable to the Nervous System | 0.52(0.34-0.80) | 0.59(0.38-0.92) |
| Symptoms Referable to the Cardiovascular and Lymphatic Systems | 0.94(0.52-1.70) | 0.69(0.38-1.28) |
| Symptoms Referable to the Eyes and Ears | 0.08(0.01-0.58) | 0.13(0.02-0.94) |
| Symptoms Referable to the Respiratory System | 1.04(0.77-1.42) | 0.94(0.68-1.29) |
| Symptoms Referable to the Digestive System | 0.50(0.36-0.69) | 0.61(0.43-0.86) |
| Symptoms Referable to the Genitourinary System | 0.42(0.24-0.71) | 0.65(0.38-1.14) |
| Symptoms Referable to the Skin, Nails, and Hair | 0.26(0.11-0.63) | 0.38(0.15-0.93) |
| Symptoms Referable to the Musculoskeletal System | 0.27(0.18-0.42) | 0.44(0.28-0.68) |
| Other | 0.42(0.30-0.61) | 0.74(0.49-1.12) |
| Is this visit related to |  |  |
| Injury/trauma | Reference [1] | Reference [1] |
| Overdose/poisoning | 0.65(0.09-4.72) | 0.56(0.08-4.10) |
| Adverse effect of medical/surgical treatment | 10.92(6.89-17.29) | 6.58(4.07-10.64) |
| Visit not related to any above | 3.29(2.34-4.61) | 2.71(1.84-4.01) |
| Questionable injury status | 3.04(0.73-12.69) | 2.35(0.54-10.14) |

Note: the adjusted OR was from a logistic regression including all variables in the table.
